# Synthesis of Xenes: physical and chemical methods

**DOI:** 10.1039/d4cs00999a

**Published:** 2025-01-23

**Authors:** Alessandro Molle, Junji Yuhara, Yukiko Yamada-Takamura, Zdenek Sofer

**Affiliations:** a CNR-IMM, Unit of Agrate Brianza via C. Olivetti 2 Agrate Brianza I-20864 Italy alessandro.molle@cnr.it; b Graduate School of Engineering, Nagoya University Nagoya 464-8603 Japan; c School of Materials Science, Japan Advanced Institute of Science and Technology (JAIST) Nomi, Ishikawa 923-1292 Japan; d Department of Inorganic Chemistry, University of Chemistry and Technology Prague Technická 5 166 28 Prague 6 Czech Republic zdenek.sofer@vscht.cz

## Abstract

Since the debut of silicene in the experimental stage more than a decade ago, the family of two-dimensional elementary layers beyond graphene, called Xenes or transgraphenes, has rapidly expanded to include elements from groups II to VI of the periodic table. This expansion has opened pathways for the engineering of elementary monolayers that are inherently different from their bulk counterparts in terms of fundamental physical properties. Common guidelines for synthesizing Xenes can be categorized into well-defined methodological approaches. On the one hand, bottom-up methods, such as physical epitaxial methods, enable the growth of monolayers, multilayers, and heterostructured Xenes. On the other hand, top-down chemical methods, including topotactic deintercalation and liquid-phase exfoliation, are gaining prominence due to the possibility of massive production. This review provides an extensive view of the currently available synthesis routes for Xenes, highlighting the full range of Xenes reported to date, along with the most relevant identification techniques.

## Abstract 2

The synthesis of 2D Xenes and related materials (*e.g.* heterostructures and hybrids) is reviewed with respect to physical and chemical methods, covering different epitaxial schemes, topotactic deintercalation and exfoliation techniques.

## Graphical abstract

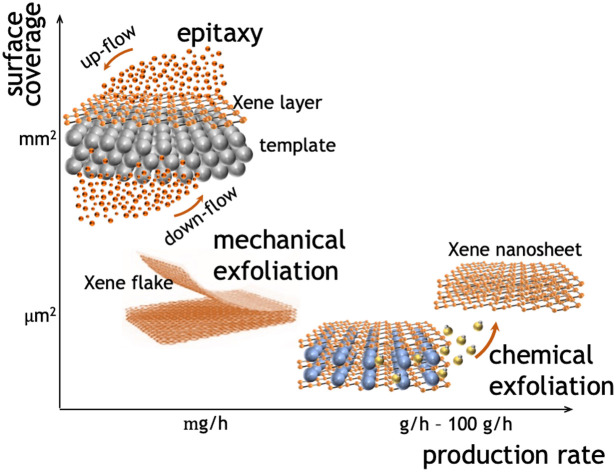

## 1. Xenes: single-element, two-dimensional materials beyond graphene

**Fig. 1:**
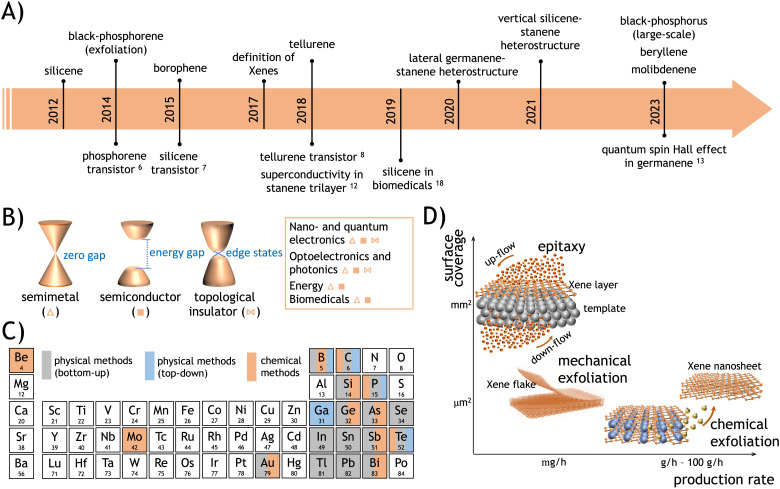
(A) Roadmap of the Xenes, including the debut of each Xene (on top of the arrow) and the technological and scientific achievements (on the bottom of the arrow). (B) Band structures classified into three groups: semimetal (△), semiconductor with a gap (■), and topological insulator (⋈). (C) List of elements in the periodic table that can be configured as Xenes, with one or more listed methods. (D) Graph of the Xene production methods *vs.* production yield and areal coverage.

The production of silicene as a reproducible experimental scheme for synthesis dates back to 2012, when an Ag(111) surface exposed to a silicon atomic beam^1–3^ and the surface segregation of Si atoms on single-crystalline ZrB_2_(0001) films grown on Si(111) wafers^4^ were thoroughly characterized, and the Si-based monolayer-thick honeycomb structures with a commensurate relationship to the underlying substrate surface were observed. This monolayer was identified as an individual two-dimensional (2D) material, termed silicene, due to its analogy with the more widely studied graphene. However, silicene exhibits distinct structural features that differentiate it from graphene. Specifically, silicene sheets, synthesized by the above-mentioned methods, can be classified as (a) an elementary layer that chemically differs from its bulk counterpart and (b) an epitaxial layer resulting from directionally oriented growth on substrates. In graphene, carbon atoms are arranged in a perfectly flat configuration, albeit with long-range ripple modulations. Atomic buckling, alternating up and down atomic positioning in a periodic pattern across the entire sheet, characterizes silicene. It turns out to be a common feature for most of the elementary 2D materials or transgraphenes that have flourished in the past decade.^5^ This class of materials has been denoted as Xenes, where X has been progressively revisited to incorporate an increasing number of elements from the periodic table, spanning metalloids from group III to group VI and, more recently, including the alkaline earth element Be and the transition metal Mo. The roadmap for Xene identification is illustrated in Fig. 1A. This is paralleled by a sequence of technological and scientific breakthroughs, including the realization of (black) phosphorene-, silicene-, and tellurene-based transistors,^6–8^ the application of silicene nanosheets as antitumoral vectors,^9^ and the first observation of a quantum spin Hall effect in germanene.^10^ This evolution is grounded in the wide variability of electronic states that the Xenes may offer, spanning from semimetallic and metallic states to semiconducting and topological insulating ones (see Fig. 1B). Such a large variety of electronic structures provides a fertile background for applications in technological fields such as nanoelectronics, quantum technologies, optoelectronics and photonics, energy technologies, and biomedicine and theranostics (see Section 5 for more details). Examples of applications in these respects include transitors,^6–8^ photodetectors,^11^ superconductors,^12^ topological quantum devices,^13^ anodes for lithium-ion batteries and supercapacitors,^14–16^ catalysts for molecular dissociation reactions,^17^ and antitumoral vectors.^18,19^ A detailed overview of the currently known Xenes, along with their respective production methods, is presented in Table 1 and pictorially summarized in Fig. 1C.

**Table 1:** List of Xenes and their synthetic methodologies according to the groups in the periodic table: type of Xene, method and scheme of synthesis (as reported in Sections 2 and 3), substrate for epitaxial schemes, and pristine source for chemical derivation from bulk or powder material. Acronyms: AE = alkaline earths, TM = transition metals, NM = noble metals, (v) = vertical, (l) = lateral, Phys = physical methods in Section 2; Chem = chemical methods in Section 3; epi = epitaxial; BP = black-phosphorus; HOPG = highly oriented pyrolitic graphite; G = graphene

Group	Xene	Method	Scheme	Substrate [temperature]	Source
AE	Beryllene	Chem	Liquid exfoliation		Be powder^20^
TM	Molybdenene	Chem	Liquid exfoliation		MoS_2_ and graphene^21^
NM	Goldene	Phys	Exchange intercalation + selective etching		Ti_3_SiC_2_
					Ti_3_AuC_2_^22^
III	Borophene	Phys	Deposition (epi)	Ag(111)^23,24^	
				Ag(110)^25^	
				Ag(100)^26^	
				Cu(111)^27^	
				Au(111)^28^	
				Al(111)^29^	
				Ir(111)^30^	
	Gallenene	Phys	Solid–melt exfoliation	GaN, Si, GaAs^31^	
	Indiene/indenene	Phys	Intercalation (epi)	SiC(0001)^32^	
	Thallene	Phys	Deposition and segregation (epi)	Si(111)^33^	
IV	Silicene	Phys	Deposition (epi)	Ag(111)^1^	
				Ir(111)^34^	
				ZrB_2_(0001)^35^	
				ZrC(111)^36^	
				Ru(0001)^37^	
				HOPG^38^	
				Pb(111)^39^	
				MoS_2_^40^	
				G/6H-SiC(0001)^41^	
				α-Al_2_O_3_(0001)^42^	
				CaF_2_/Si(111)^43^	
			Segregation (epi)	ZrB_2_(0001)/Si(111)^4^	
			Intercalation (epi)	G/Ru(0001)^44^	
				hBN/ZrB_2_(0001)/Si(111)^45^	
		Chem	Topotactic deintercalation	Sr,Gd,Eu/Si(111)^46–48^	CaSi_2_^49^
	Germanene	Phys	Deposition (epi)	Au(111)^50^	
				Pt(111)^51^	
				Al(111)^52^	
				MoS_2_^53^	
				Cu(111)^54^	
				AlN/Ag(111)^55^	
				Sb(111)^56^	
				HOPG^57^	
				Ag(111)^58,59^	
			Segregation (epi)	Pt/Ge(110)^60^	
				Ag(111)^61^	
				Al(111)^62^	
				Ag_0.9_Al_0.1_(111)^63^	
				Au(111)^64^	
			Intercalation (epi)	MoS_2_^65^	
		Chem	Topotactic deintercalation		CaGe_2_^66^
	Stanene	Phys	Deposition (epi)	Bi_2_Te_3_(111)^67^	
				Ag(111)^68^	
				Cu(111)^69^	
				Au(111)^70,71^	
				Sb(111)^72^	
				Pd(111)^73^	
				InSb(111)^74^	
				PbTe(111)^75^	
	Plumbene	Phys	Deposition (epi)	Fe/Ir(111)^76^	
				Au/Pb(111)^77^	
			Segregation (epi)	Pd(111)^78^	
V	Black phosphorene	Phys	Physical exfoliation		BP^79^
			Pulsed laser deposition	Mica^80^	
		Chem	Chemical exfoliation		BP^81,82^
	Epi-(blue)phosphorene	Phys	Deposition (epi)	Au(111)^83^	
	Arsenene	Chem	Chemical exfoliation		As^84^
	Antimonene	Phys. Chem	Deposition (epi)	PdTe_2_^85^	
			Chemical vapor deposition	Ge^86^	
	Bismuthene	Phys	Deposition (epi)	SiC(0001)^87^	
				Ag(111)^88^	
VI	Selenene	Phys	Deposition (epi)	Si(111)^89^	
	Tellurene	Phys	Deposition (epi)	Mica^90^	
			Self-assembling from SiTe	Sb_2_Te_3_^91^	
		Chem	Chemical exfoliation (hydrothermal synthesis)		Te-I^92^
Hetero-structures	Graphene/silicene (v)	Phys	Intercalation (epi)	Ru(0001)^44^	
	MoS_2_/silicene (v)	Phys	Deposition (epi)	MoS_2_^40^	
	Graphene/germanene (v)	Phys	Deposition and segregation (epi)	Ag(111)^93^	
	Stanene/germanene(l)	Phys	Deposition and segregation (epi)	Ag(111)^94^	
	Silicene/stanene (v)	Phys	Deposition (epi)	Ag(111)^95^	
	Stanene/silicene (v)	Phys	Deposition (epi)	Ag(111)^95^	
	hBN/silicene (v)	Phys	Intercalation (epi)	ZrB_2_(0001)/Si(111)^96^	

Nowadays, it is apparent that diverse methodologies can be implemented to synthesize Xenes by design, and these generally encompass physical and chemical methods depending on whether Xene formation is dictated by a physical process (nucleation and growth or mechanical cleavage) or is mediated by a chemical reaction or treatment. The former one mainly consists of diverse epitaxial schemes as a bottom-up approach for Xene growth. We also add some notable examples of top-down exfoliation of Xene nanosheets from bulky crystals, such as the case of (black-) phosphorene from black phosphorus (BP),^97^ which can also be considered a physical method. The chemical route mainly involves topotactic deintercalation from layered compounds, liquid exfoliation enabled by chemical solvents, or thermally induced phase separation.

The different approaches to Xene synthesis are schematically illustrated in Fig. 1D as a function of the surface coverage scale and the production rate, defined as the areal extent that is uniformly wet by a single-layer or multilayer Xene and the rate at which the weight of an individual Xene is produced for a given synthesis method, respectively. Generally, epitaxial methods, as a bottom-up approach to crystal growth, result in a superior control of the surface structure gained from the atomic control of the growth evolution. In addition, the epitaxial methods are effective in gaining a large-area coverage experimentally demonstrated up to cm^2^ scale but virtually extendable to the wafer scale depending on the spatial constraints affordable in the ultra-high vacuum (UHV) production condition. The overall yield in terms of massive production is quite limited, and the cost per production item is generally high owing to the use of the UHV equipment. Conversely, chemical methods may yield cost-effective massive production with micro-scaled flakes eventually embodied in a framework, thus leading to a considerably larger production yield but scarce control of the structural features in the obtained framework of nanosheets. Exfoliation (either physical through mechanical cleavage, for instance, or chemical through solution-induced delayering) as a top-down approach to the derivation of Xene nanosheets is limited by the extraction of microscaled flakes with good-to-high structural quality, low cost, low yield, no scalability, and availability of the host layered materials. Further details on scalability and production issues are discussed in Section 4.

From this background, we review Xene synthesis methodologies. Unlike other reviews on the topic, our focus here is on the synthesis schemes enabling the isolation of Xenes on substrates or as freestanding membranes, and all along this way, how they can be engineered and/or adapted to fabricate new materials (*e.g.* heterostructures) or optimize the existing ones and to enable technology transfer to a functional application.

## 2. Physical methods for Xene Synthesis

Physical methods for Xene synthesis encompass bottom-up and top-down approaches. The former one is concerned with epitaxial crystal growth, whereas the latter one mainly consists of an exfoliation scheme that does not involve the mediation of chemical reactions.

**Fig. 2:**
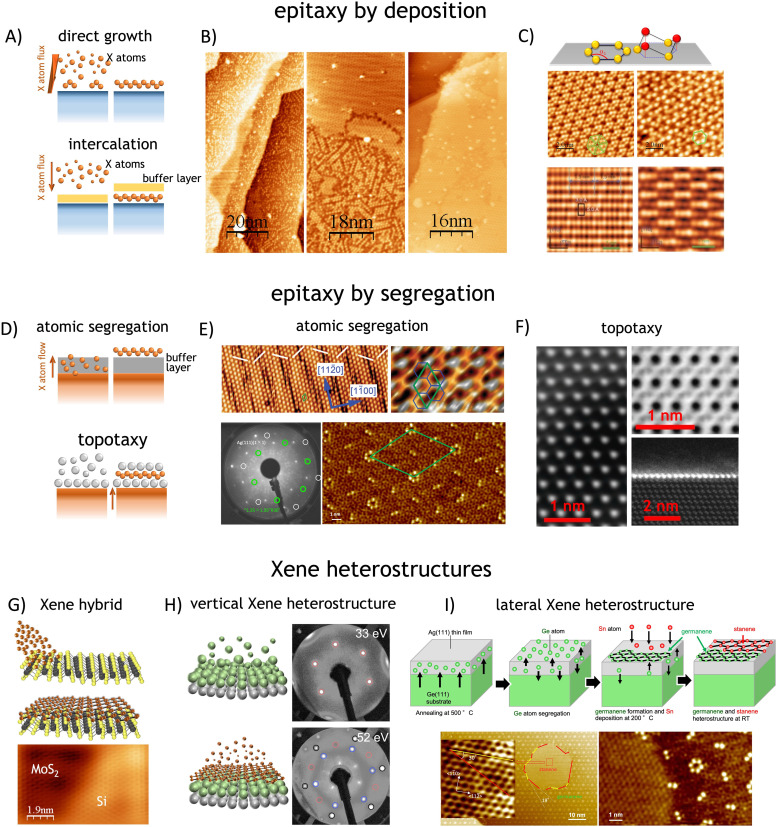
Physical methods. *Xene epitaxy by deposition*: (A) simplified sketches of the direct growth and intercalation-mediated growth. (B) Evolutive scanning tunneling microscope (STM) images of the silicene formation by direct growth on Ag(111) at 0.1 ML, 0.3 ML, and 1 ML coverage (from left to right), adapted from ref. 98 (with permission from IOP). (C) Non-planar Xene structures: buckled phases in silicene (4 × 4) and 
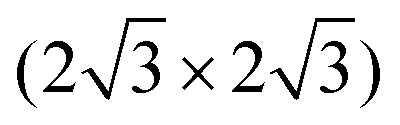
 phases indicating the coincidence relationship with the surface unit cell), adapted from ref. 98 (with permission from IOP), and β_12_ and χ_3_ phases of borophene, adapted from ref. 99 (with permission from Springer-Nature). *Xene epitaxy by segregation*: (D) simplified sketches of the segregation through a buffer layer and topotaxy. (E) STM images (with two different scales) of silicene spontaneously grown by segregation from a Si substrate through a (0001)-oriented ZrB_2_ buffer layer (top), adapted from ref. 4 (with permission from APS through a Creative Commons Attribution 3.0 License) and LEED pattern with STM image of germanene segregation from Ag(111) thin film on Ge(111) substrate (bottom), adapted from ref. 61 (with permission from IOP). (F) Scanning transmission electron microscope images of silicene layer in CaSi_2_ formed by segregation from a Si substrate and topotactic reaction with a Gd overlayer. Adapted from ref. 47 (with permission from Springer-Nature): top-view (left), cross-section view of bulk and monolayer GdSi_2_ (right); *Xene heterostructures*: (G) Xene and transition metal dichalcogenide (TMD) hybrid heterostructure: MoS_2_/silicene with Si grown on a MoS_2_ surface, adapted from ref. 40 (with permission from Wiley). (H) Silicene–stanene heterostructures by sequential epitaxy on Ag(111), representative sketches (left) and corresponding LEED patterns (right), based on results from ref. 95. (I) Growth of lateral germanene-stanene heterostructure by atomic segregation from a Ge substrate through a (111)-terminated epi-Ag layer that is partially covered by stanene domain grown by direct growth according to the process schematics (top) and the atomically resolved topography of the stanene domain laterally surrounded by germanene (bottom), adapted from ref. 94 (with permission from Wiley).

Epitaxy is a bottom-up approach for producing an overlayer on the precisely oriented surface of a given substrate. Etymologically, the term comes from the Greek roots “epi” and “taxis,” meaning “above” and “order”, respectively, and thus refers to the growth of an ordered single crystal above a substrate serving as a template. Thus, the epitaxial character of a crystal is reflected in the monocrystalline nature of the grown layer consistent with the normal direction of the growth surface. In this framework, diverse (physical) epitaxial schemes can be implemented,^5^ which span from the bare molecular beam deposition (where the X atom flux impinges on the growth surface through a vacuum environment) to the top-to-bottom intercalation (where the deposition is mediated by a buffer layer on the growth surface) to the bottom-to-top segregation (where the X atoms are supplied by the substrate through a buffer interface layer); see Fig. 2 for an overview of the different schemes. An essential point for all the approaches is the role of the template in Xene's growth. A template, namely the surface of the epitaxial substrate, exhibits an atomic lattice that is commensurate with an energetically admitted Xene structure; namely, the template and the Xene surface unit cells have the least common multiple. Finally, echoing the concept of the van der Waals heterostructures, we pay attention to the particular case of epitaxial Xene heterostructures, where different Xenes are vertically or laterally interfaced. A list of the epitaxial methods and related experimental cases is given in Sections 2.1–2.3. Section 2.4 is dedicated to physical exfoliation schemes adapted to specific cases.

In parallel, let us also mention that Xenes derived by physical methods are usually addressed by a characteristic number of experimental techniques that are markedly surface-sensitive and mainly operate under UHV conditions (namely, the environment where the Xenes are usually grown).^100^ Scanning tunneling microscopy/spectroscopy (STM/STS) and atomic force microscopy (AFM) are utilized to obtain detailed information on the atomic-level resolution of surface structures and spatially local electronic properties. The STM images are obtained through a tunneling current reflecting a local electronic structure near the Fermi energy. Thus, it is crucial to examine whether the STM images have any sample bias voltage dependence for geometrical determination from the STM images. AFM images are obtained by attractive or repulsive force between the tip and the surface, thus directly reflecting the atomic position. For both methods, knowing the structural relationship between Xene and the substrate is usually tricky unless a Moiré structure is observed in the STM images. Low-energy electron diffraction (LEED) is convenient for examining the uniformity and symmetry of Xene (2, 3, 4, or 6 fold) and the structural relationship between Xene and the substrate, such as azimuthal rotation angles and the ratio of lattice constants (commensurate or incommensurate). Reflection high energy electron/positron diffraction (RHEED/RHEPD) and surface X-ray diffraction (SXRD) are also powerful tools for identifying the symmetry of the geometrical structure and its height from the substrate. Auger electron spectroscopy (AES), X-ray photoemission spectroscopy (XPS), and core-level photoemission spectroscopy (PES) provide a coverage or compositional ratio as well as an impurity analysis. PES and Raman spectroscopy are used to examine the local bonding and crystal structure, respectively. Raman spectroscopy is a powerful tool for identifying silicene and germanene because of the specific peaks observed, similar to graphene. In this respect, Raman spectroscopy allows for discriminating the single layer and multilayer regime in terms of a red shift due to a tensile strain and a blue shift due to compressive strain^101^ and for precisely determining the thickness of the Xene sheets from the relative separation between the main Raman spectral features, such as in the case of the B_2g_ and A_2g_ modes of exfoliated BP flakes.^102^

Furthermore, angle-resolved photoemission spectroscopy (ARPES) provides an valence band structure near the Fermi energy. Usually, these experimental techniques are paralleled and supported by *ab initio* calculations based on density functional theory (DFT), which allows for the interpretation and rationalization of the experimental data.^103^ In particular, following a conventional surface science analytical approach, the theoretical modeling serves as a throughput to the interpretation of experimental data, *e.g.* in the identification of the atomic structure and local density of states from STM/STS measurements (see, for example, ref. 23 and 99 for the case of borophene), the electronic band structure is close to the Fermi level from ARPES measurements (see, for example, ref. 69 and 104), and the characteristic vibrational modes from Raman spectra.^105^

### 2.1 Deposition

Xenes can be grown by (1) *direct deposition of X atoms in vacuo* on a supporting substrate, or (2) *intercalation* of the evaporated X beam, *e.g.* in between a buffer layer and the substrate.

#### 2.1.1 Direct deposition

Nucleation and crystal growth induced by adatoms, *i.e.*, adsorbed atoms from a vapor phase on a directionally oriented surface, are the pivotal mechanisms of deposition-driven epitaxy according to a general physical vapor deposition (PVD) scheme. They may occur upon direct exposure to the molecular beam (direct growth), see Fig. 2A. Molecular beam epitaxy (MBE) qualifies as a peculiar PVD case, where the evaporation flux travels in a ballistic regime as no scattering occurs in the UHV environment of the growth. Currently, most of the available Xenes are obtained following this methodology with the supporting substrates working as a commensurate template to stabilize the Xene. Suitable substrates enabling the Xene growth comprise (a) the (111)-plane of face-centered cubic metals, such as Ag,^1,23,24,58,59,68,88^ Cu,^27,54,69^ Au,^28,50,70,71,83^ Al,^29,52^ Ir,^30,34^ Pd,^73^ Pt,^51^ and Pb;^39^ (b) the (0001) plane of layered compounds, such as MoS_2_ and Bi_2_Te_3_;^40,67^ (c) the (111) or (0001) plane of large gap semiconductor or insulator crystals, such as SiC (3C, 4H, and 6H)^32,41,87^ and sapphire (α-Al_2_O_3_).^42^ The case of Ag(111) is historically interesting because it was the first substrate enabling the epitaxial growth of silicene^1^ owing to the characteristic hybridization of the Si and surface Ag orbitals.^106^ The growth evolution of silicene on Ag(111) is displayed in Fig. 2B starting from the sub-monolayer coverage where filamentary chains nucleate on the surface to a progressive coalescence of surface domains to complete large-area wetting.^98^ Therefore, Ag(111) offers a pretty general platform for the Xene epitaxy as demonstrated by the relevant cases of the silicene (as mentioned above), stanene, borophene, antimonene, and bismuthene (see Table 1), namely the 2D counterparts of tin (from the Latin name “stannum”), boron, antimony, and bismuth, respectively. The physical reason for the compliance of the Ag(111) substrate (and other metal substrates with similar termination) with many Xenes relies on the commensurability between the substrate surface lattice and the energetically admitted structures of the Xenes, *i.e.*, a proportionality between the lattice unit cell of the substrate surface and the Xene structure of minimum energy. This relationship determines the details of the Xene atomic arrangement. Consequently, the so-grown Xene can be recast as (a) an ultra-flat layer or as (b) a buckled layer with one or more registries (*i.e.*, surface phases or superstructures) (see Fig. 2C). In type (a), Xenes usually grow as an ultra-flat (*i.e.*, unbuckled) layer. This is the case of stanene and bismuthene grown at low temperatures on Cu(111) and Ag(111), respectively,^68,69,88^ where the interaction with the substrate selects the orbital composition around the Fermi level.^107^ In type (b), buckling stems from the non-planar dislocation of the X atoms in a vertically distorted hexagonal lattice. It is usually subject to allotropism of the surface phase owing to the structurally allowed multiple configurations. For instance, the epitaxy of silicene on Ag(111) can result in several surface phases conventionally denoted in terms of the coincidence periodicities with respect to the theoretically relaxed (substrate-free) silicene and to the Ag(111) surface lattice, *e.g.*
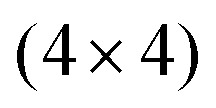
, 
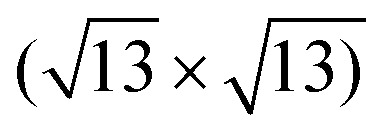
, and 
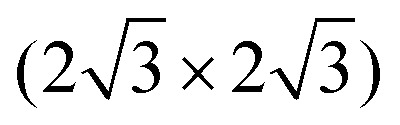
,^1,108–110^ see Fig. 2C. The alternation of these surface phases is a rate-limited process dictated by growth kinetics, *e.g.*, by varying the substrate temperature during growth. Diverse silicene phases can coexist although each phase proportion is temperature-dependent.

In contrast, single-phase selection can be obtained by Ag surface engineering *via* atomic Sn decoration and buffering.^111^ Stepping forward to the second and multiple layers leads to the emergence of a unique type of surface order, denoted as the 
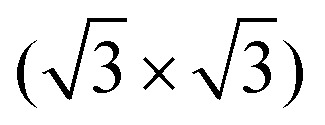
, in a three-dimensional (3D)-like growth mode, namely with the topmost domains nucleating before the underlying layers are completed. This reconstruction was interpreted either as an intrinsic silicene feature or as a superstructure of segregated Ag atoms through a diamond-like silicon film. The two pictures were reconciled within the argument of temperature-dependent activation of Ag atom segregation^101,112^ according to which silicene grows multilayer only in a narrow temperature window where Ag diffusion is hindered.

Another example of allotropism is borophene (2D boron) as epitaxially grown on Ag(111). In the MBE scheme, the boron atoms are thermally evaporated onto the substrate temperature at a given temperature ranging from nearly 300 °C to 380 °C to avoid clustering.^99,113^ Taking Ag(111) as a representative substrate again, two ordered phases qualify the growth of borophene, which correspond to the β_12_ and χ_3_ model structure of borophene conceptual structure, see Fig. 2C (bottom).^114^ Based on first-principles calculations, borophene is made possible by removing B atoms from the pristine triangular flat sheet, thus creating an atomic vacancy.^115^ This event can, therefore, generate a handful of possible 2D lattice configurations quantitatively identified by the global structural parameter *h* (*i.e.*, the ratio between vacancy sites and the available atomic site in a unit cell) and the coordination number *c* for each B atom in the lattice. The two experimentally observed phases coincide with (*h*,*c*) = (1/6,5) and (*h*,*c*) = (1/5,4) for the β_12_ and χ_3_ structures, respectively.^99^

Metal surfaces may also serve as catalyzers for the growth of Xenes. This is the case of the so-called blue phosphorene, namely a 2D allotrope of phosphorus with a rhombohedral structure at variance with the more popular black phosphorene with an orthorhombic structure, where the host Au(111) surface selectively facilitates the dissociative absorption of the P_4_ molecular beam, supplied by the sublimation of a BP matrix in the crucible of a Knudsen cell. The so-dissociated P atoms self-assemble on a herringbone-reconstructed Au(111) surface with a flower-like motif assigned to the Au-P framework model, where substrate Au atoms decorate the blue-phosphorene domain.^83,116^ More recently, within the same scheme of direct deposition from the vapor phase, epitaxial phosphorene was grown on Cu(111), thereby providing a large-area coverage.^117,118^ The epitaxy of blue phosphorene was initially regarded as the path to bypass the limited scalability of the mechanically exfoliated black phosphorene from BP. However, recently, large-area production of black phosphorene was reported using pulsed laser deposition.^80^ Using a similar method, Xene growth was demonstrated on non-metallic substrates serving as templates. Among these are 2D layered crystals, as in the case of the silicene or germanene growth on MoS_2_^40,53^ or stanene growth on Bi_2_Se_3_,^67^ or single-crystal large-gap crystals, as in case of the growth of silicene on α-Al_2_O_3_(0001),^119^ graphene/SiC(0001),^41^ CaF_2_/Si(111),^43^ and bismuthene on SiC(0001).^87^ Recently, in addition to the 2D-layered sheets, 1D- nanoribbon of silicene was synthesized on Ag(110) surface.^120^ The geometrical structure is determined to be a pentagon-related structure, which is theoretically proposed to induce *p*-wave superconductivity and is expected for the emergence of spin-polarized Majorana zero-modes.^121^

#### 2.1.2 Intercalation

Intercalation of the evaporated atoms through a buffer layer can also promote the formation of the Xene layer between the buffer layer and the substrate. This method is closely related to the growth of a hybrid Xene heterostructure.^122^ In these heterostructures, the Xene is coupled with another 2D material of a different type, as long as the buffer layer frequently consists of a 2D layer where intercalation is made possible through intrinsic structural defects. This case is further elaborated at the end of Section 2.3 in terms of the Xene heterostructure. Although a buffer layer made of a 2D material prevents the formation of covalent bonding with the X atoms due to the inherent lack of dangling bonds, an artificially created non-2D buffer layer may work for the same purpose without jeopardizing the assembly of the Xene through interaction. Within this fashion, Zhang *et al.* reported the synthesis of monolayer blue phosphorene by silicon intercalation on Au(111) by dealloying blue-phosphorene-Au compound and then forming a gold silicide buffer layer.^123^ Similarly, the tellurium layer on Au(111) also provides growth of quasi-free-standing blue phosphorene.^124^ Among the intercalation schemes, we also mention liquid gallium intercalation through epitaxial graphene (namely graphene-on-SiC), which results in an embedded gallenene layer.^125^ Stabilization of a liquid phase is typical of gallium at room temperature, and it has been exploited for the top-down production of gallenene, as reported in Section 2.4. More recently, indium atoms based on epitaxial graphene on SiC(0001) were shown to undergo intercalation through the graphene layer (at 500 °C, followed by post-deposition annealing), thereby reconfiguring as an indenene or indiene layer.^32^

### 2.2 Segregation

At variance with the direct deposition through MBE, Xene epitaxy can occur the other way around through X atoms diffusing from the substrate to the surface, namely by segregation from the substrate with or without the presence of a buffer layer (Fig. 2D). An example of this mechanism is the so-called atomic segregation epitaxy (ASE), which involves fewer experimental parameters than deposition methods. Basically, only three ingredients are mandatory within the ASE scheme of operation: the substrate X supplying the X atoms through a buffering layer, the buffer layer serving as a template for the desired Xene growth, and careful control of the substrate temperature (see Fig. 2D and E). In this framework, the temperature of the sample can be uniformly controlled, thus making it relatively straightforward to fabricate highly crystalline 2D materials on an ultra-large scale and benefiting from the segregation flux. The pristine sample, consisting of the X substrate with the buffer layer, is heated at temperatures that are relatively higher than those in the deposition scheme (*e.g. T* > 645 °C for silicene on ZrB_2_(0001)/Si(111),^126^*T* > 480 °C for germanene on Ag(111),^61^ and *T* > 430 °C for germanene on Al(111)^62^), thereby triggering atomic (X) segregation to the very surface level where the hot (mobile) X atoms self-organize according to the crystalline match with the buffer layer. In this method, the X atoms melt in bulk at high temperatures or diffuse through crystal defects in the buffer layer, such as grain boundaries. Therefore, the most energetically stable 2D materials can spontaneously be prepared on UHV-treated substrates. A pioneering study in this respect was carried out in 2012 by Fleurence *et al.* during the research process, in which atomic layer silicene was fabricated on metallic zirconium diboride (ZrB_2_) thin films epitaxially grown on Si(111) by ASE; see Fig. 2E top panel.^4^ The lattice constant of silicene has a magic mismatch to that of ZrB_2_(0001). This is a critical aspect of the epitaxial growth as long as lattice matching induces strong bonding between silicene and ZrB_2_, which may affect the properties of free-standing silicene. Nevertheless, the ASE method has demonstrated the size of the grown silicene up to the cm^2^ scale, which can be made even larger depending on the design of the growth system.

In the ASE method, lattice matching is important for fabricating a single-crystal film on a crystal substrate. Ag crystals are one of the best candidates for lattice matching with Ge crystals, with a lattice constant ratio of 3 : 4. Experimentally, Ag(111) thin films with high crystalline quality were grown on Ge(111).^127–129^ In 2018, germanene synthesis using the ASE method was reported for Ag(111) thin films on the Ge(111) substrate.^61^ The lattice size of germanene is close to free-standing germanene,^130^ and the lattice matching between germanene and Ag(111) is extremely large, with a length of 5.35 nm, corresponding to a 
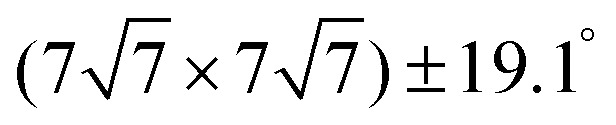
 superstructure (see Fig. 2E, bottom panel). The germanene prepared by ASE is significantly different from the germanene prepared by MBE, even on the same Ag(111) surface. This is mainly because the relatively lower sample temperature is restricted to prevent the dissolution of Ge atoms into Ag(111) in the MBE method.^58,131,132^ Specific phonon modes in germanene have been identified by tip-enhanced Raman spectroscopy (TERS) combined with STM imaging.^133^ Subsequently, graphene-capped Ag(111)/Ge(111) samples were also used for interfacial germanene synthesis, which was also confirmed by Raman spectroscopy.^93^ Recently, structural models of germanene with 
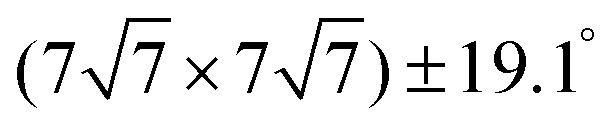
 and 
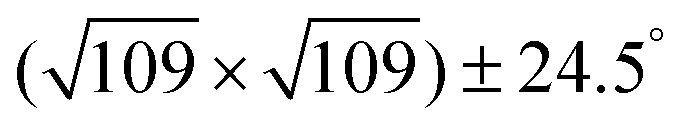
 reconstructions on Ag(111) are proposed using STM, surface X-ray diffraction, and DFT calculations.^59^

Because the unit cell sizes of Au and Al are similar to those of Ag, Al(111) and Au(111) films with high crystalline quality have also been fabricated on Ge(111). Ge atoms in the Ge(111) substrate indeed dissolve into the Al and Au thin films and segregate on the Al(111) and Au(111) surfaces. For Al(111) thin films, segregated Ge atoms form germanene with a (2 × 2) superstructure matching with the Al(111)(3 × 3) unit cell,^62^ which is identical to the direct deposition case. In the latter method, there are two germanene phases of (2 × 2) and 
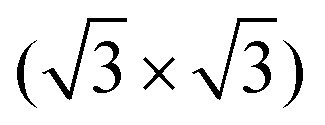
 with very similar Ge coverages,^134^ resulting in a mixed phase with the same layer. Conversely, the ASE method readily leads to a (2 × 2) single phase.^62^ For Au(111) thin films on a Ge(111) substrate, segregated Ge atoms form (5 × 8) reconstruction, which is slightly different from germanene prepared by direct deposition epitaxy.^50,135^ In 2019, with a similar approach, plumbene was synthesized using a Pb_*x*_Pd_1−*x*_ alloy (111) surface on Pd(111).^78,136^ Pd is one of the best elements for Pb surface segregation because Pb–Pd alloys are thermally very stable at temperatures higher than 1200 °C for the Pb_*x*_Pd_1−*x*_ alloy (*x* < 0.12).

Another approach to segregation-assisted epitaxy of Xenes is the topotaxial growth of silicene and germanene embedded in Zintl compounds MX_2_ (where M is a rare earth element such as Eu or Gd, or Sr, and X is Si or Ge) on Si or Ge substrates, respectively. In detail, a pre-deposited nanoscale M film encounters segregation of the X atom from the substrate and, under tailored constraints, reacts with the X atom by forming the layered MX_2_ compound where the X atoms self-arrange in a planar sheet between two top and bottom M layers^46–48^ (see Fig. 2F). Thus, the so-functionalized Xene sheet is affected by the surrounding environment. Consequently, owing to magnetic anisotropy or dipolar interactions, it displays a magnetic order, *e.g.*, ferromagnetism, at the 2D level.

Although not directly associable with segregation schemes, as reported above, it is worth mentioning the self-assembling approach to the epitaxial growth of α-tellurene results from the Te atomic chains that are released from SiTe grown on a Sb_2_Te_3_ template under non-equilibrium conditions.

### 2.3 Epitaxial growth of heterostructures

We can classify two types of heterostructures: vertical and lateral heterostructures. The former type may appear as hybrid and pure Xene heterostructures. Hybrid Xene heterostructures incorporate a Xene layer grown on a 2D layered non-Xene substrate (single or multilayered) template by benefitting from van der Waal epitaxy or selective hybridization at the surface. However, a pure Xene heterostructure displays a sequence of two different Xene layers. Relevant cases in this respect are stanene on Bi_2_Te_3_, and silicene or germanene on MoS_2_.^53,67,137^ In both cases, the Xenes display a regularly buckled structure in which the lattice constant coincides with that of the substrates with an overall metallic character.

Within the hybrid Xene heterostructures, Chiappe *et al.* reported the growth of 2D Si nanosheets on MoS_2_ surfaces examined by STM and STS measurements supported by DFT calculations, ending up in silicene with a metallic character^40^ and bending of the MoS_2_ electronic bands^138^ (see Fig. 2G). On the other hand, silicene layers on MoTe_2_, GaS, and GaSe are predicted to be gapless semiconductors, with preserved Dirac cones at the *K* points.^139^ Other similar systems, such as germanene on MoS_2_,^53,65^ antimonene on PdTe_2_,^85^ silicene nanosheets on graphite,^38,140^ and germanene islands on graphite,^57^ were also intensively studied. The cases of Ge and Si deposition on MoS_2_ were reconsidered in terms of Ge and Si atom intercalation through the MoS_2_ layer.^65,141^ The intercalation path can be driven by thermally activated crossing through structural defects in the layered substrate; thus, it depends strictly on the quality of the MoS_2_ substrate. Similarly, Sn deposition on TiS_2_ was examined by STM because Sn atoms are intercalated mainly from the step edge.^142^ The structural defects and interstitial atoms in the layered TiS_2_ substrate affect the intercalation migration process.

For a vertical heterostructure, a stable silicene-graphene heterostructure is prepared on Ru(0001) by silicon deposition on graphene/Ru(0001), as well as a silicene-hBN monolayer heterostructure on ZrB_2_(0001) film on Si(111), with intercalation of Si atoms in between graphene or hBN monolayer on substrates.^41,44,45^ Li *et al.* observed that graphene plays the role of a capping layer for silicene.^44^ Wiggers *et al.* demonstrated by core-level photoemission spectroscopy that the hBN monolayer prevents oxidation of underlying silicene at least for an hour, acting as a capping layer despite its monolayer thickness.^45^ Titter *et al.* used a few graphene layers as a capping layer to encapsulate silicene.^143^ Within the same vertical heterostructure scheme, Dhungana *et al.* demonstrated the stacking of silicene on stanene and *vice versa* by sequential epitaxial growth on a Ag(111) substrate, thereby paving the way to an artificial pile-up of diverse Xenes on a superlattice structure (see Fig. 2H).^95^

For the lateral heterostructure, the first attempt was reported by Kiraly *et al.* in 2015, in which the sequential deposition of carbon and silicon on Ag(111) resulted in the synthesis of both lateral and vertical graphene–silicon heterostructures.^144^ Graphene–borophene heterostructures are also demonstrated on Ag(111) using a similar method, reporting on the synthesis of both lateral and vertical interfaces.^145^ The first achievement of a true lateral heterostructure with epitaxial Xenes was provided by Ogikubo *et al.* in 2020 in which germanene-stanene lateral heterostructures are synthesized on Ag(111) using the combination of MBE and ASE epitaxies.^94^ (see Fig. 2I).

### 2.4 Physical exfoliation

Top-down approaches are concerned with Xenes being exfoliated, extracted, or reduced from a bulky phase instead of being atomically grown and self-organized on a substrate, and may involve either physical operations (*e.g.* mechanical cleavage) or chemical treatment (*e.g.* liquid phase exfoliation). Thus far, they include (black) phosphorene (a single layer of black phosphorus), gallenene, tellurene, and beryllene (see Table 1). Mechanical exfoliation is the pristine way graphene and other consolidated 2D materials were extracted from bulky crystals. This was also the case of phosphorene, originally referred to as the 2D single-layer derived from a black-phosphorus crystal, namely the phosphorus allotrope displaying an orthorhombic structure in a layered solid. Multilayered phosphorene (from a single layer to a few nm) was initially derived through mechanical exfoliation using scotch tape adhesion to bulk black-phosphorus crystals. The so-obtained flakes were integrated into a transistor device structure with *p*-type conductivity (as illustrated in the roadmap in Fig. 1A).^6^

Taking advantage from the liquid metal character of gallium at room temperature, atomically thin gallenene (2D gallium) sheets were obtained by a solid–melt interface exfoliation method consisting of a stamp of a Ga droplet, kept at a temperature slightly higher than its melting temperature, onto a SiO_2_/Si wafer brought into contact with the surface of the Ga droplet.^31^ The lower temperature at the SiO_2_–Ga interface results in the solidification of the surface Ga layers, thus effectively leading to the exfoliation of an mm-scaled sheet from the melt.

## 3. Chemical methods for Xene synthesis

**Fig. 3:**
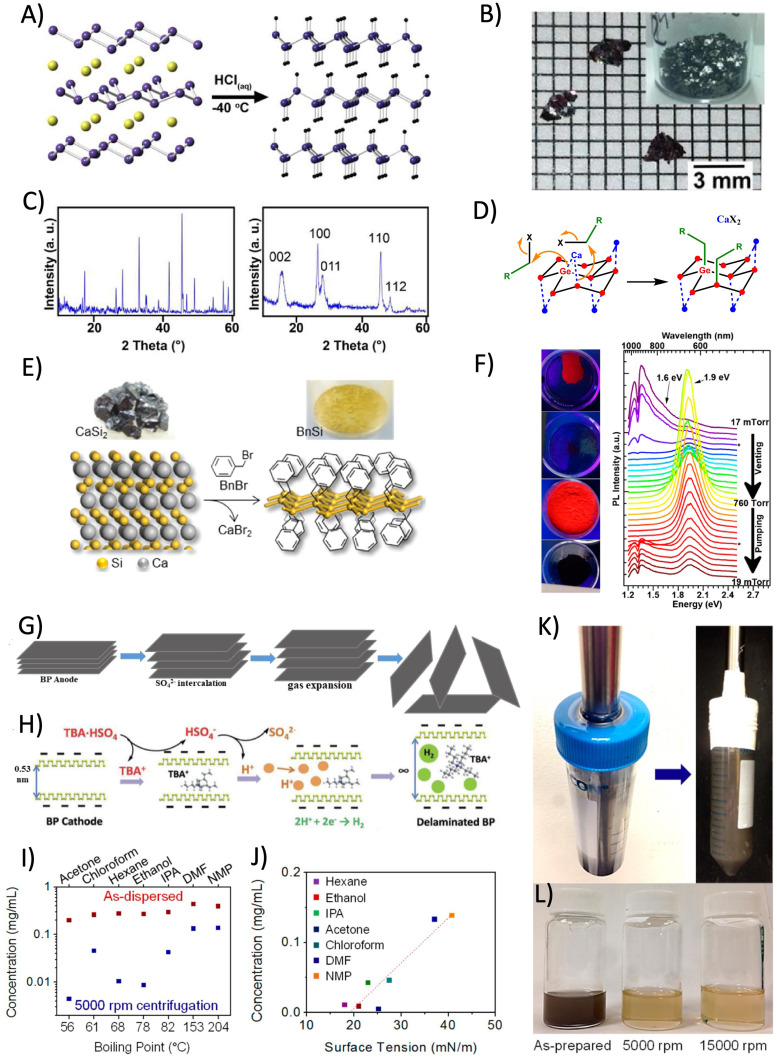
Chemical methods. *Topotactic deintercalation.* (A) Reaction scheme of calcium-selective etching from CaGe_2_ Zintl phase by hydrochloric acid. (B) Flakes of germanane obtained from CaGe_2_. (C) X-ray diffractogram of CaGe_2_ before and after chemical exfoliation. (Reprinted from ref. 66 under permission from ACS). (D) Reaction mechanism of CaGe_2_ exfoliation and functionalization by alkylhalogenides. (Reprinted from ref. 146 under permission from ACS). (E) Scheme of benzylsilicene synthesis by reaction of calcium disilicide with benzylbromide. (Reprinted from ref. 147 under permission from ACS). (F) Photoluminescence of methylated germanene with and without the presence of moisture and corresponding photoluminescence spectra. (Reprinted from ref. 148 under permission from ACS). *Liquid-phase exfoliation*. Electrochemical exfoliation of BP to phosphorene using anodic exfoliation (G) and cathodic exfoliation using (H) tetrabutylammonium hydrogensulfate. (Reprinted from ref. 149 under permission from Elsevier). Ultrasonication setup for black phosphorus under an inert atmosphere (I). Photo of dispersion in NMP before and after centrifugation (J). Concentration of exfoliated BP in various solvents (K). Dependence of concentration on solvent surface tension (L). (Reprinted from ref. 150 under permission from ACS).

The top-down approaches in tetrel chemistry covering silicene and germanene, as well as the huge spectra of their derivatives, are well known. Indirect methods based on chemical exfoliation, so-called topotactic reaction or deintercalation, are applied to compounds containing hexagonally arranged layers of silicon and germanium, which resemble those of Si(111) or Ge(111) bilayers from bulk silicon or germanium crystallized in the diamond structure. These compounds, known as a Zintl phase with the general formula of MX_2_, where M is typically calcium or europium, and X is silicon or germanium in the form of the hexagonally packed layer with in-plane bonds, are key precursors for topotactic (also named topochemical) conversion to silicene/germanene and their derivatives. Topotactic or topochemical reactions are generally chemical processes that lead to chemical changes without significant structural changes in the material. Zintl phase conversion to *silicene/germanene* is an example of such a chemical process. This type of Zintl phase has been known for decades, and the existence of CaSi_2_ was already reported in the middle of the 19th century, together with its topotactic conversion. The silicon-germanium alloy forms a continuous solid solution system where germanium can also be substituted up to about 25 at% tin. The topochemical deintercalation of calcium layers from calcium disilicide, calcium digermanide, and their solid solution were reported using different etchant and electrochemical methods, typically providing covalent functionalized silicene and germanene derivatives.^49^ The synthesis of non-functionalized silicene and germanene is much rarer, and such materials are extremely reactive because covalent functionalization leads to the stabilization of the 2D pocked honeycomb structure. The mechanism of CaGe_2_ chemical exfoliation together with formed flakes is shown in Fig. 3A and B.^66^ Chemical exfoliation is nicely documented by X-ray diffraction, where calcium digermanide is at low temperature by reaction with acid converted to hydrogentated germanene (germanane) with significantly higher lattice spacing (Fig. 3C).

Chemically derived Xenes differ from epitaxial Xenes in terms of characterization methods and are commonly investigated by several material characterization techniques; see, for example, ref. 130,132. The methods of analysis depend strongly on the composition of the Xenes. In general, XPS is used to determine the surface chemical composition and can be combined with energy (EDS) or wavelength dispersive spectroscopy (WDS) alongside various electron microscopy methods. However, significantly greater information depth provides more “bulk-like” information about elemental composition, but extreme spatial resolution of chemical composition can also be achieved. Electron microscopy-related methods with outstanding spatial resolution (especially transmission microscopy in combination with EDS or electron energy loss spectroscopy (EELS) provide information with extremely high lateral resolution theoretically down to single atoms but obtained informations about chemical bonds are limited. XPS has limited spatial resolution because it works with X-ray as an excitation source. However, high spectral resolution allows for obtaining detailed information about the bonds of individual atoms. The XPS method is crucial for Xene surface chemistry analysis, including the identification of the type and degree of surface functionalization as well as the type of bonds between Xene and surface atoms. This method is highly complementary to the vibration spectroscopy method, as infrared spectroscopy can provide complex information about surface chemistry. Valuable informations are obtained by vibrational spectroscopy methods, namely infrared (FT-IR) and Raman spectroscopy, where tip-enhanced and related techniques can provide information with lateral resolution even beyond the diffraction limit, such as tip-enhanced Raman spectroscopy (TERS), scanning near-field optical microscopy (SNOM) and related techniques. As said for the case of mechanically exfoliated BP flakes, Raman spectroscopy is practical in readily determining the thickness from the separation of the characteristic vibrational modes, *e.g.* the case of the A^1^ and E^2^ modes in tellurene.^151^ For infrared spectroscopy, various methodologies are employed depending on the absorption properties of the analyzed material (such as diffuse or specular attenuated total reflectance, or optical transmittance methods). In particular, the diffuse reflectance (DRIFT) method can provide valuable information even for highly absorbing samples in a broad spectral range using dilution with KBr and measuring temperature dependence by operando high-temperature cells. The FT-IR method is complementary to Raman spectroscopy, which offers a high spatial resolution. However, in Raman spectroscopy the information about chemical modification is more limited than that obtained from the infrared spectroscopy methods. Beyond these well-known methods, other techniques, such as combustion elemental analysis, thermal analysis combined with evolved gas analysis, or solid-state nuclear magnetic resonance (NMR), can provide valuable insights and a more detailed view of particular aspects of material chemistry and surface functionalization. For Xenes prepared by applying top-down methods, X-ray diffraction provides crucial information about phase composition, the number of layers in the system, and lattice parameters. The layer distance is strongly influenced by the functionalization group, and these effects can be clearly resolved by X-ray diffraction. Surface morphology analysis is performed using various probe and electron microscopy methods.

Although electron microscopy (scanning electron microscopy and transmission electron microscopy) provides valuable information about morphology and the associated methods give information about material composition, direct thickness obsevation and estimation the number of layers requires probe microscopy methods. AFM is generally used to obtain information about flake thickness distribution, while conductive and Kelvin force methods provide additional information about surface chemistry and electronic properties. Compared to “bottom-up” physical methods, STM is not broadly used for characterization of exfoliated Xenes due to surface defects and contaminants originating from “top-down” chemical methods.

In what follows, we address specific examples of topotactic deintercalation with a focus on silicene, germanene, and recent advances in other Xenes (Section 3.1), and then we consider alternative chemical approaches to Xene synthesis (Section 3.2) and the case of liquid-phase exfoliation (Section 3.3).

### 3.1 Topotactic deintercalation

The most common layered Zintl phase, CaSi_2_, has been well known for many decades and is in a low-purity form used in tone scales in the steel industry. However, the germanium-based counterparts are significantly more novel, and most of the reports originate from the 21st century. The first report on the topotactic formation of the silicene derivatives originated from German chemist Friedrich Wöhler in 1863, who reported the reaction of CaSi_2_ with cold aqueous hydrochloric acid to form a yellow powder currently known as planar polysiloxene, a silicene derivative with the general formula Si_6_H_3_(OH)_3_,^152^ as follows:3CaSi_2_ + 6HCl + 3H_2_O → 3CaCl_2_ + Si_6_H_3_(OH)_3_ + 3H_2_ (*T* ∼ 20 °C)3CaSi_2_ + 6HCl → 3CaCl_2_ + Si_6_H_6_ (*T* ≪ 0 °C)This reaction was explored and studied in detail in subsequent years, and Kautsky resolved its structure^153–158^ and systematically studied its properties. Acid exfoliation at low temperatures and in non-aqueous solvents led to a hydrogen-terminated surface, highly reactive silicane.^159^ Many derivatives of silicene beside hydrogen-terminated surfaces have been reported since Kautsky's work was published.

Additionally, pure silicene can be synthesized by the topotactic conversion of calcium disilicide. Pure silicene synthesis was reported using the reaction of CaSi_2_ with SbCl_3_, producing silicene together with antimony and calcium chloride,^160^ as follows:3CaSi_2_ + 2SbCl_3_ → 3CaCl_2_ + 6Si + 2SbOther methods for silicene formation are based on reactions with selected acids as well as high-temperature reactions with HCl and others.^161^ Halogen-terminated silicene has been reported by several authors since the 1960s. The etching of CaSi_2_ with ICl and IBr produces chlorine and bromine-terminated surfaces.^162^ Halogen derivatives of silicene are highly reactive and can be converted to other silicene derivatives. The reaction with hydrides, such as LiAlH_4_, led to a hydrogen-terminated surface, while the reaction with fluorination reagents, such as SbF_3_, produced a fluorine-terminated surface. Halogen-terminated surfaces can also react with organometallic compounds, including alkyl and aryl lithium compounds, as well as with Grignard reagents. This opens possibilities for the flexible functionalization of the silicene surface. CaSi_2_ can also directly react at high temperatures with amine salts, such as methylammonium bromide, forming Si–NH–CH_2_ bonds.^163^ This reaction proceeds to almost complete functionalization of silicene, where only sterically complex amines produce a lower degree of functionalization. Recently, methods have also been reported based on the direct reaction of CaSi_2_ with alkyl halide. This reaction led to the formation of calcium dihalide and the formation of Si-C bonds, producing various alkyl-functionalized silicene. The mechanism of calcium disilicide (and digermanide) is shown in Fig. 3D and E.^146,147^ Similar to other topotactic reactions, the removal of reaction byproducts can be crucial for reaction progress, and a phase transfer reagent for the removal of calcium halide can significantly help the reaction progress. The same method was used to synthesize benzylsilicene (Fig. 3E). Silicane can also be used as a starting material. For example, direct reaction with Grignard reagents led to the formation of Si–C bonds.^164^ Organic functionalization of silicane significantly improves its stability towards oxygen and moisture and can be stored in air for several days without significant changes of its chemistry and increase of oxygen content. Additionally, amines can react with silicane, forming Si–N–C covalent bonds. This method was used to introduce various hydrocarbons to silicane surfaces.^165^ Usually, the degree of functionalization is very high and is limited only sterically. Organic diamines can be used to restack silicane, where the interlayer spacing can be clearly correlated with the length of the diamine.^166^

Compared to silicene and its derivatives, the chemistry of germanene and its derivatives is less investigated. The first topotactic deintercalation of calcium from the CaGe_2_ structure was reported in 1944.^167^ The first synthesis of hydrogenated germanene, which is called germanane, was reported by Goldberger significantly later in 2013.^66^ The strategy used for synthesis is identical to the methods applied to silicane based on low-temperature etching with aqueous hydrochloric acid according to the following reaction:CaGe_2_ + 2HCl → 2GeH + CaCl_2_ (*T* < 0 °C)Compared to silicane, germanane is significantly more stable and can be handled in air. Depending on the structure of the starting calcium germanide, the formed germanane can adopt different stacking arrangements, either 1T or 6R.^168^ Besides the synthesis of germanane, other reactions were reported with calcium germanide for the synthesis of alkyl germanene derivatives. CaGe_2_ can react with alkyl halide following the formation of various derivatives of germanene and calcium dihalide. This type of reaction was first reported by Goldberger *et al.* using a CH_3_I reaction with calcium germanide forming *methylgermanene*.^169^ The exfoliation of CaGe_2_ by various alkyl halides, including benzyl halide derivatives, was further explored by other groups, who showed differences in the reactivity of alkylhalogenides containing chlorine, bromine, and iodine.^170^ The crucial point for the direct reaction of the Zintl phase is the effective removal of the reaction byproduct (calcium halogenide), which has typically limited solubility in alkylhalogenides and aprotic solvents. Usually this is achieved using a porous mechanical separator (*e.g.* by fritted glass) between the organic phase with the Zintl phase compound and aqueous phase.^170^ Another approach is using a solvothermal system with a mixture of organic solvent, alkylhalogenide and water as described by Liu *et al.*^171^ Formed alkyl derivatives of germanene are moisture sensitive, and co-intercalation of water, *e.g.*, from atmospheric humidity, led to significant changes in its electronic structure, which is documented by significant changes in photoluminescence properties (Fig. 3F).^148^ CaGe_2_ can also be halogenated similarly to the approaches reported for the synthesis of silicene halogen derivatives, and reaction with ICl led to the highly reactive germanene with iodine termination, which can be further substituted with organometallic reagents, including a less reactive Grignard reagent. This method can effectively introduce direct covalent bonds between the aromatic system and germanium,^146^ which are impossible to induce by the reaction of CaGe_2_ with arylhalogenide. The other effective method for the functionalization of germanene is based on the reactivity of hydrogenated germanene*–germanane*. Hydrogen in germanane can be effectively substituted either by the formation of a negative charge on the germanene skeleton using strong electron donors (either using liquid NaK alloy or alkali naphthalenide), which create a negative charge on the germanene skeleton and can be effectively substituted by alkyl halide.^172^ Recently, there were also reported methods using the direct reaction of germanane with butyllithium to produce *butylgermanane* as a germanene derivative.^173^ These methods show high flexibility of reactions using germanane as a starting material compared to Zintl phase compounds. The various aspects of germanene derivative synthesis by applying top-down methods were compared by Hartman *et al.*,^174^ showing significant differences not only in the degree of functionalization but also in the oxidation of the germanene skeleton by site reactions, especially in water-based systems. Electrochemical exfoliation and functionalization were also reported for the synthesis of functionalized germanene and its solid solutions with silicon. Electrochemical decalcification of CaGe_2_ and CaGe_*x*_Si_(2−*x*)_ gives a significantly low degree of functionalization compared to chemical methods, and dominantly, only the edges of sheets are functionalized.^175^

*Borophene* is the only other Xene prepared by topotactic reactions. Similar to Zintl phases, many diborides with AlB_2_ structure consist of honeycomb layers of boron atoms with metal atoms arranged in between, with the most well-known example MgB_2_. The topotactic exfoliations of MgB_2_ and other diborides were reported by several groups. Zhang reported the use of I_2_ in acetonitrile-producing Mg_0.22_B_2_ nanosheets.^176^ Additionally, sonication experiments produce Mg deficient borophene-like structures.^177^ Chemical removal of cations led similarly to silicene/germanene chemistry to hydrogen-terminated surfaces. The chemical etching of Mg from MgB_2_ has been reported to produce hydrogen-terminated borophene.^178^ Chemical etching has also been reported for different borides, such as YCrB_4_, to produce hydrogen-terminated borophene nanosheets.^179^ Cationic resins are used by several groups to bind cations during selective topotactic etching to enhance the speed of conversion. Compared to the tetrel group, borophene and its derivatives possess significantly higher reactivity, and a significantly lower number of publications have been reported on the topotactic formation of borophene and its derivatives.

### 3.2 Thermal deintercalation and other chemical methods

Zintl crystals, such as CaSi_2_, are a good platform for deriving silicene by thermally decoupling the Ca from the silicene plane upon differential thermal treatment. This approach relies on the different boiling temperatures of Ca (1484 °C) and Si (2900 °C) constituting planes so that silicene planes can already be de-alloyed from CaSi_2_ at 900 °C.^180^ A thermally induced transformation was recently proven to be the mechanism for the nucleation of molybdenene (2D molybdenum) from MoS_2_ powder.^21^ In detail, molybdenene was synthesized by microwave exposure of a mixture of MoS_2_ and graphene, where graphene serves as a heat absorption catalyst at the interface with MoS_2_. The so-generated microwave power melts MoS_2_ (loosen bonds) when the temperature reaches its melting point (*T*_m_ = 1185 °C) and results in Mo–S bond breaking with the release and migration of Mo atoms towards the self-organization of molybdenene whiskers. Released Mo atoms (rich in electrons) respond to such an enormous electric field and migrate through the graphene–MoS_2_ mixture, constituting a molybdenene layer.

Although broadly used in semiconductor science and technology, there are few cases of chemical vapor deposition (CVD) of Xenes on selected substrates that can accommodate both the local Xene nucleation and the pyrolysis of the metal–organic molecular precursor involved in the process. In this respect, antimonene multilayered nanocrystals were grown on a (111)-terminated Ge substrate by benefitting from the surfactant effect of Au nanoparticles on the surface and concomitantly exploiting the SbCl_3_ precursor dissociation and Cl-induced surface etching.^86^

### 3.3 Liquid-phase (or chemical) exfoliation

Exfoliation can be carried out in liquid using appropriate solvents, reagents and conditions. Liquid-phase or chemical exfoliation has gained interest so far as a method for the cost-effective and scalable production of 2D materials. In this respect, under ultra-sonication, phosphorene was exfoliated by rinsing in selective solvents, usually *N*-methyl-2-pyrrolidone.^181^ A similar approach has been recently adopted for the exfoliation of beryllene (2D beryllium), namely upon dispersion of dimethylformamide (DMF) solvent, sonification, centrifugation, and thermal drying in a nitrogen environment.^20^ No other way is currently reported for the synthesis of beryllene flakes. In addition to mechanical exfoliation, electrochemical-assisted exfoliation is well known for several van der Waals Xenes. This method was mainly applied to black phosphorus-producing high-quality single- and few-layer phosphorene with high aspect ratio.^182^ Owing to its sensitivity towards oxidation, exfoliation is typically performed in an organic solvent under an inert atmosphere. The electrochemical and mechanical exfoliation are shown in Fig. 3G–L. Electrochemical exfoliation can be performed using an anodic method (Fig. 3G) and a cathodic exfoliation method (Fig. 3H). Mechanical exfoliation was demonstrated for several Xenes. In particular, black phosphorus solvent-assisted mechanical exfoliation has been reported by many authors. Because Xenes are highly sensitive to oxygen and moisture, exfoliation is usually performed in dry organic solvents under a protective atmosphere (Fig. 3I), followed by size selection of exfoliated materials by centrifugation (Fig. 3J). The yield of solvent-assisted exfoliation strongly depends on the solvent's physical properties, such as its surface tension and boiling points (Fig. 3K and L). Topotactic reactions are also applicable to several other Xenes. From the pnictogen group, among the allotropes of phosphorus (arsenic, and antimony) adopting the layered structure, direct exfoliation can be performed either using solvent-assisted methods such as share force milling and ultrasonication or more gentle processes of electrochemical exfoliation, which by intercalation can weaken the layer bonds and provide exfoliated sheets with a significantly higher aspect ratio compared to mechanical exfoliation methods. The synthesis of goldene (2D gold) by chemical exfoliation was also reported recently. It is based on the substitution of silicon in the Ti_3_SiC_2_ MAX phase (with M, A, and X being a transition metal, a group A element, and C or N, respectively) by gold and the subsequent selective etching of titanium and carbon.^22^ This method is suitable for the production of flakes with lateral sizes exceeding 100 nm stabilized by organic surfactants. Within chemical exfoliation processing, we may consider tellurene as derived by the so-called hydrothermal synthesis that was originally developed to synthesize one-dimensional (1D) tellurium nanostructures.^183^ In this scheme, sodium telluride is added to a solution of polyvinylpyrrolidone (PVP). After dissolution, hydrazine and ammonium hydroxide solution are added as a reducing agent and for pH adjustment, respectively, and the temperature is increased up to 180 °C, thus resulting in dissociated tellurium flakes with a trigonal phase in the solution that can be fished and adapted to substrates. Compared to the 1D tellurium synthesis, the case of 2D tellurene nanosheets is different because the pristine material ratio is carefully adjusted and the reaction time is elongated adequately to yield single crystal 2D flakes with lateral sizes; over 100 μm and thicknesses ranging from monolayer to tens of nm can be obtained.

## 4. Technological viability

For most Xenes, the research scope is limited to a niche investigation because of the lack of viable integration protocols in a technology flow. This is why Xene production should be paralleled by a concomitant effort to develop production and processing schemes that enable the transfer of Xene technology. This section is intended to discuss the technological potential of Xenes in terms of the rich electronic properties (Section 4.1), the path to Xene production standards (Section 4.2), limiting issues in Xene handling (such as the lack of stability under environmental conditions) and related solutions of processing and applications (Section 4.3), and the step towards integration with a future outlook on Xene-related emerging technologies (Section 4.4).

### 4.1 Salient electronic properties

**Fig. 4:**
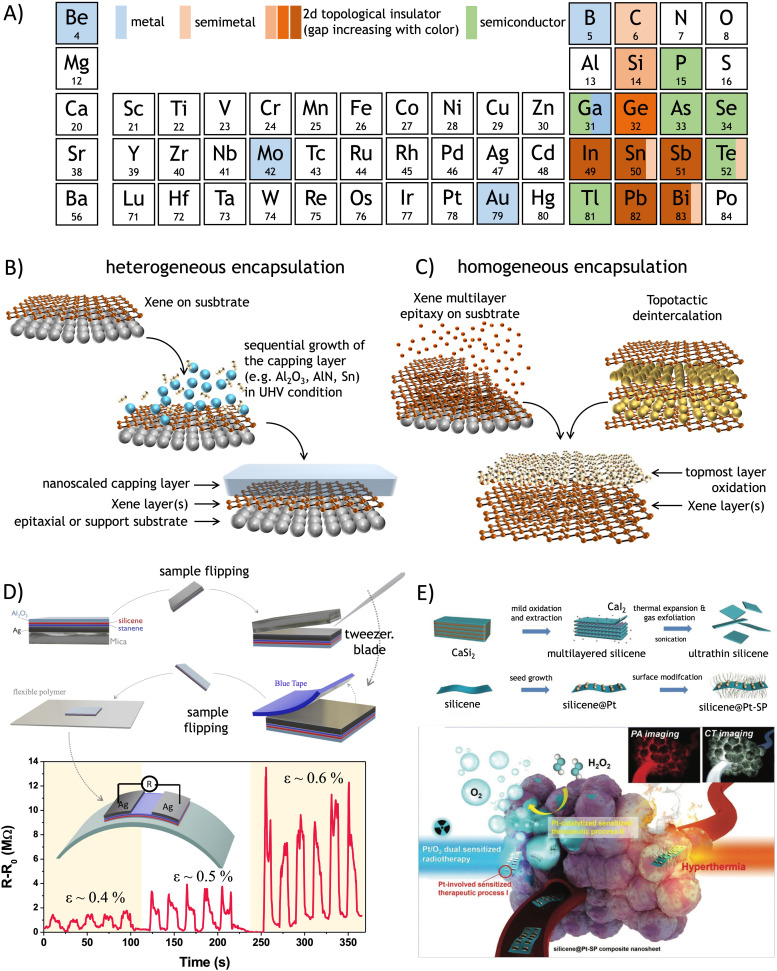
(A) List of the Xenes in the periodic table with the relevant electronic character. (B) Scheme of heterogeneous encapsulation starting from the Xene accommodated on a substrate, and the sequential deposition of a capping layer with nanoscale thickness. (C) Scheme of homogeneous encapsulation resulting from the oxidation of an Xene multilayer derived from epitaxy (left) or from topotactic deintercalation (right). Recent cases of processing and applications of the Xenes after heterogeneous and homogeneous encapsulation. (D) Delamination of silicene from an epitaxial silicene/stanene heterostructure on Ag(111) ending up with an all-around encapsulated membrane to be deposed on a secondary target substrate for the integration into a piezo-resistor device where the resistivity is varied as a function of the applied bending force *ε* (expressed as a ratio of the radial deformation and the membrane thickness in %). Adapted from ref. 192 (reprinted with permission from Wiley). (E) Topotactic deintercalation of silicene nanosheets from CaSi_2_ through mild oxidation, sonication, and subsequent functionalization with Pt surface nanoparticles towards the exploitation for theranostic application for the hyperthermal treatment of the tumors. Adapted from ref. 193 (reprinted with permission from Wiley).

Before entering the details of Xene processing, it is worth paying attention to those physical and chemical properties that distinguish synthetic Xenes from other nano-scaled materials and make them suited for specific target applications. One of the salient features of the Xenes in all their forms and configurations is to display an extremely rich variety of electronic states spanning from metals, to semimetals (either topological or not), 2D topological insulators, and semiconductors. Although the electronic character may vary in the same Xene as a function of the surrounding conditions (*e.g.* substrate, interface, strain, doping, and capping), to a general view, Xenes can be classified as in Fig. 4A. In detail, several Xenes present a non-trivial topology. These include Xenes in the group IV where, according to the Kane–Mele model,^184^ the spin–orbit coupling interaction in the Hamiltonian causes the opening of an energy gap state in the Xene body and the emergence of topologically-protected edge states.^185,186^ This effect is negligible at relatively light atomic mass, namely in graphene, silicene, and germanene (with gaps of 1, 10, and 20 meV, respectively), which behave as semimetals at room temperature, but it becomes sizeable when the atomic mass becomes larger, namely in stanene and plumbene (with a gap of up to 0.42 eV).^187^ This “topological strength” is expressed with an increasingly darker brown color in Fig. 4A. The same topological character can be found in other “heavy” Xenes, such as bismuthene and antimonene.^188^ Interestingly, non-trivial topological phases of different physical origins can be induced in the 2D-to-3D transition of the epitaxial film growth. For instance, the α-phase of tin, which recasts as stanene at the single-layer level, becomes a Dirac semimetal or topological insulator in the bulk form depending on the induced epitaxial strain (compressive or tensile, respectively) when grown on InSb.^189^ Tellurene is a different case in the topological framework. It appears as a semiconductor with a thickness-dependent energy gap (from 1.0 eV in the single-layer to 0.3 eV in the multi-layer),^190^ but topological Dirac cones are incorporated in its conduction band, and they bear a quantum Hall effect owing to Weyl fermion under extrinsic *n*-type doping.^191^ Borophene and recently discovered molybdnene, beryllene, and goldene are metallic, whereas gallenene may have an ambipolar metallic-semiconducting character.^31^ Other known Xenes (thallene, phosphorene, arsenene, and selenene) are semiconducting.^33,79,84,89^ Clearly, the electronic character concurs with determining the target application(s) of each Xene along with the configurational layout, namely the form in which the Xene is released for an application function.

### 4.2 Scalability and massive production

Chemical and physical methods for the Xene synthesis have been developing rapidly in the last decade, and a portfolio of Xenes has been prepared using these same methods. Besides the most well-known group IV elements (tetrels) and group V elements (pnictogens), new members of this family have been reported in the last decade, including tellurene from group VI and borophene from group III elements. These approaches are likely extended in the future to other elements where only theoretical simulations are available for now (see, for instance, the concept of aluminene^194^). Note that alkali metals from group I were experimentally found to form highly-buckled honeycomb structures when intercalated in between bilayer graphene.^195^ The limiting factor of industrial use in the real world is the scalability of several Xenes for synthesizing bulk materials, such as BP, or its high price (such as germanium). However, recent progress on the pulsed laser deposition of BP holds promise regarding production scalability.^196^ In some applications, such as electronics, only a minute amount of active material is needed which is compatible with a Xene monolayer coverage (corresponding to a fraction of a milligram per square centimeter). Conversely, for bulk applications such as energy storage, Xene cannot be massively supplied on a large scale and quantity owing to their production cost, complications in the synthesis, and limited availability. An example of these constraints is represented by the case of BP. This material has been known for more than a century, but the scalable methods of its synthesis are still unknown, and the synthesis of batches over 10 g has not been reported in the literature. However, this material can be produced by high-pressure synthesis like diamonds, and it can find its place in the market, especially in electronic and other high-tech applications. The Xenes limitation by price originating from environmental abundance is associated with germanium, with current market prices significantly exceeding 3000 USD per kilogram. This fact poses a disadvantage for bulk applications, such as energy storage, because its price exceeds more than ten times that of other materials currently commercially used in batteries and supercapacitors. However, we have very cheap and broadly abundant materials, such as boron and silicon with enormous application potential in rechargeable batteries. The 2D structure in these cases can help solve several problems associated with current boron and silicon-based anodes for Li-ion batteries, such as structural breakdown upon electrode expansion and limited conductivity.^197^ This approach is promising for applications with a massive amount of material to cope with printed electronics, energy storage fields, catalysis, or environmental remediation.

In Table 2, we report on a comparative analysis among the synthesis methods described in Sections 2 and 3 in terms of surface coverage (defined as the amount of surface area that a Xene in a single-layer or multilayer form wets when it is posed on a substrate), production rate (defined as the weight of the produced Xene in the time unit), atomic control (defined as the capability to tailor the Xene structure at the atomic level either in terms of the structural order or atomic modifications), and production cost (qualitatively defined as the effort for the production of an individual Xene sample).

**Table 2:** Comparative analysis for the synthesis methods reported in Sections 2 and 3 in terms of surface coverage, production rate (in gram or milligram per hour), atomic control, and production cost

Methods	Production scheme	Surface coverage	Production rate	Atomic control	Production cost
Physical	Deposition/segregation/heteroepitaxy	mm^2^ to cm^2^	mg h^−1^	Achievable	High
	Exfoliation	μm^2^	mg h^−1^	Not reported	Low
Chemical	Topotactic deintercalation/dealloying	μm^2^	1–10^2^ g h^−1^	Possible	Moderate
	Liquid phase exfoliation/hydrothermal synthesis	μm^2^	mg h^−1^	Not reported	Low

Compared to graphene, the growth of mono- or multilayer Xenes by chemical methods and liquid-phase exfoliation (as reported in Section 3) is still under development, and only limited area growth has been reported so far, thus limiting production scalability by the moment. This fact originates primarily from the huge variability in the chemical and physical properties of the Xenes family. For example, to appreciate striking differences within the Xenes, tellurium has very low redox potential, and its reduction from a positive oxidation state is easily achievable using broad spectra of reducing reagents, while the very high redox potential of boron or silicon caused such synthesis to be very challenging. Owing to this variability in the chemical properties, specific solutions should be designed for each Xene case. In terms of production rate, chemical methods for Xene synthesis give a production rate of the order of 1 to 100 g h^−1^ (gram per hour) in the case of topotactic deintercalation and continuous vapor-dealloying from CaSi_2_.^161,198^ The chemical process for Xene synthesis can be engineered to have atomic modifications by design, but the approach is still at an early stage. However, flakes with a microscaled size are the usual product of top-down exfoliation schemes. In this case, the structural quality of flakes is high with basically the lowest cost possible, but the scalability is quite poor and the atomic control is not possible within the exfoliation handling.

According to Sections 2 and 3, the bottom-up synthesis of Xenes can proceed either by gas phase/molecular beam depositions or by chemically assisted synthesis. Epitaxial methods, including deposition and segregation, generally result in large-scale surface coverage on the cm^2^ area, thus preluding to a wafer scalability of the epitaxial Xene production schemes within the spatial constraints of the growth apparatus (*e.g.* the manipulator in MBE systems, samples holders in CVD reactors). Large-scale uniformity can be hurdled by grain formation (for instance, due to multiple phases) or by substrate-related constraints (*e.g.* the width of the substrate surface terraces), leading to linear and point defects, but surface treatments can be developed to optimize the atomic structure and the growth process can be engineered to incorporate local modifications, such as dopant atoms. However, the same methods can generally benefit from structural control at the atomic level in the Xene layer (either in terms of large-area lattice uniformity or atomistic modification like doping), which is beneficial for technology applications such as nanoelectronics, spintronics, and quantum technologies, where a superior structural quality is a stringent requisite. In this respect, strategies for Xene layer optimization or atomistic modification are still under development for reliable standardization and for the viable exploitation of Xenes in operational devices. Such an atomic control, however, is not counterbalanced by a sufficiently high production rate to face massive application of Xenes because it is usually limited by expensive schemes (*e.g.* MBE) with a quite slow growth rate. If we assume that most of the epitaxial Xenes are grown on a mm^2^-to-cm^2^ scaled area with a growth rate of a few monolayers per hour, the resulting production rate amounts to the order of a few mg h^−1^ (milligram per hour) and a production quantity of the order of mg per process batch, namely 3 to 5 order of magnitudes lower than that obtained from chemical methods. Production costs can be reduced by applying chemical methods such as topotactic deintercalation or dealloying ending up in a much larger production rate (in the range of 1–10^2^ g h^−1^) and much lower surface coverage (basically confined in the formation of flakes). A concise summary of this analysis is displayed in the diagram of Fig. 1D, where the synthesis methods are reported in terms of their efficiency on the surface coverage *vs.* production rate.

### 4.3 Stabilization, processing, and emerging applications

Unlike graphene, most other Xenes exhibit high reactivity, such as borophene, silicene, and phosphorene, and without encapsulation or chemical modification, they undergo decomposition in the air very fast, in some cases even in an order of seconds or minutes. This is a challenging factor for Xene synthesis and applications. Environmental instability results from the chemical reactivity of dangling bonds in the Xene layer due to the inherent atomic arrangement, *e.g.*, the mixed sp^2^/sp^3^ hybridization in buckled Xenes, adventitious defects causing oxidation or degradation, or moisture absorption. Air sensitivity is a severe hurdle to the technology exploitation of Xenes, and stabilization strategies strictly demand that Xene be embodied in technology. Thus far, environmental instability has been addressed using top face encapsulation schemes, including either *heterogeneous encapsulation* (Fig. 4B) *via* the sequential growth of a capping layer after Xene accommodation on a substrate (*via* deposition or exfoliation) or *homogeneous encapsulation* (Fig. 4C) by piling up multiple layers of the same Xenes (*i.e.*, Xene multilayer).^101,199^ Both kinds of encapsulation schemes allow Xenes to be preserved from degradation under environmental exposure out of the growth ambient. A homogenously encapsulated transistor based on a silicene multilayer has been proven to display a more durable operation^101^ than the rapidly degrading single-layer silicene,^7^ but the multilayer regime affects the inherent properties in the single layer owing to partial covalent bonding. On the same line of the homogeneous encapsulation, silicene nanosheets derived by topotactic de-intercalation are inherently suited to be released in solution albeit oxidation of the exposed surface layers.^200^ Although the Raman spectrum of the so-derived silicene makes evidence of a 2D hexagonal ring mode,^201^ the nanosheet thickness is uncontrollably variable, and the nanosheet surface is unavoidably subject to oxidation.

According to heterogeneous encapsulation, several options have been reported, which include Al_2_O_3_, AlN, CaF_2_, and Sn capping layers,^45,202–205^ or the post-growth printing of a graphene or hBN.^206,207^ In particular, Al_2_O_3_-based encapsulation, namely the sequential growth of Al_2_O_3_ after Xene epitaxy, was originally demonstrated to result in a chemically inert and non-interacting capping layer, which led to the observation of the Raman spectrum of silicene outside the growth ambient.^202,208^ Stabilization strategies like homogeneous and heterogeneous encapsulation are the paths to obtain and manipulate durable Xene membranes or free-standing layers. As a paradigmatic case of applications, as depicted in Fig. 4D and E, we report two different representative ways to manipulate silicene membranes from epitaxy and deintercalation for flexible electronic and biomedical applications, respectively. In the former case (Fig. 4D), silicene membranes are derived from the delamination of the epitaxial silicene and the heterogeneous encapsulation with an Al_2_O_3_ layer.^192^ The use of an epitaxial Ag(111) film on a layered mica substrate for the silicene epitaxy instead of an Ag single crystal is the enabling step for silicene delamination from the pristine substrate because of the layered character of the mica support. Indeed, mica can be readily removed by mechanical cleavage, and the overlying Ag can be etched off using I-based solutions. For the latter purpose, either wet or dry methods can be implemented with the final goal of having an Ag/silicene/Al_2_O_3_ membrane readily transferable to a secondary target (either rigid or flexible) substrate.^209^ An applicative example is shown in Fig. 4E, which displays the exploitation of the so-delaminated silicene membrane in a piezoresistive device whose channel resistance *R* is reversibly varied (from the equilibrium value, *R*_0_) by varying the applied mechanical bending (*ε*) under multiple cycles.

Removing the Ag template residue from the Xene again shows the same stability issue as the environmental exposure at the top face level (namely before encapsulation). However, the epitaxial template cannot be easily bypassed because it is structurally functional for Xene growth. Interface engineering can be a suitable solution to have one Xene buffered from the pristine template by the other Xene. This is the case with the vertical silicene–stanene heterostructure, as illustrated in Fig. 2G, where the stanene serves as a sacrificial layer after the complete etching of the Ag template, thus inhibiting the oxidation of the embedded silicene in an all-around encapsulated silicene membrane.^95^

Xenes, by topotactic deintercalation, are stabilized *via* homogeneous encapsulation induced by self-limited oxidation, and they can be effectively exploited for biomedical and theranostic applications. Indeed, they harness the photothermal conversion performance in biological environments, thus targeting tumor treatment, anticancer drug delivery, bioimaging, and tumor radiotherapy.^19,210^ Similar to other 2D nanosystems for integrating nanoparticles onto surfaces towards therapeutic or synergistic multi-functionalization, the large surface area of 2D silicene/silicon nanosheets (SiNSs) allows guest drug molecules to be either used for loading and delivery or functionalized with nanoparticles.^210^ As a representative case, silicene derived from CaSi_2_ by topotactic deintercalation and functionalized with Pt nanoparticles on its surfaces has proven to synergistically enhance the photothermal ablation (conversion)^211^ and radiotherapy of the tumor cell (see Fig. 4E).^193^ The 2D SiNSs have also been explored for Raman bioimaging application and suppression of bacterial growth.^18^

### 4.4 Integration in device platforms and future outlook on emerging technologies

**Fig. 5:**
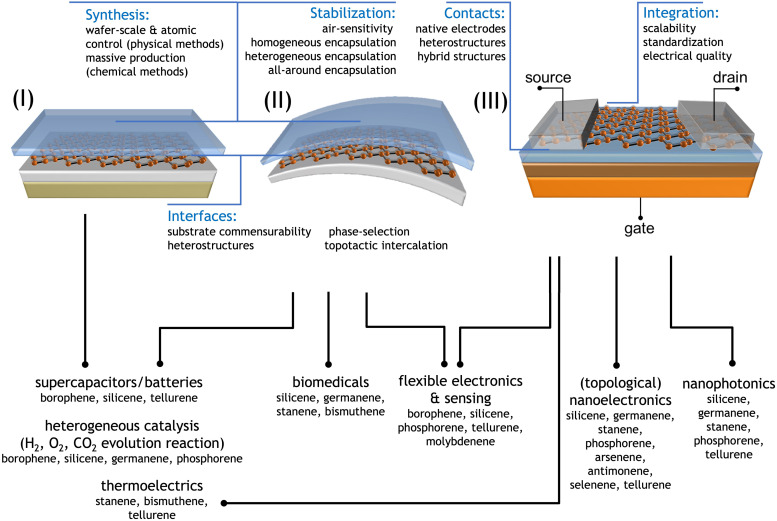
Paradigmatic Xene configurations: (I) Xene on a rigid platform, (II) Xene as a flexible membrane, and (III) Xene on a device platform. For each configuration, specific and common issues (synthesis, stabilization, and interfaces in common for I and II, reconfigurability for II, contacts and integration for (III) and main target applications are reported.

Currently, the Xene production is limited to the lab level, and its scope is mostly concerned with a surface science investigation. Configuring Xenes in technology viable layouts is essential to determine the electronic properties of the Xenes, as described in Section 4.1, and to identify target applications. In Fig. 5, we illustrate three paradigmatic Xene configurations in the latter respect: (I) Xene in a rigid platform, (II) Xene as a flexible membrane, and (III) Xene in a device platform. Each configuration is qualified by the characteristic manufacturing details. Xenes in layouts (I) and (II) are extremely sensitive to the surrounding environment, and interface or configurational engineering is effective in protecting and/or selecting the Xene structure, as mentioned in Section 2. Layout (I) is readily related to the epitaxial methods and, thus, is suited to wafer scale production with atomic control (see Sections 2 and 4.2 for the details of the experimental cases and related issues). Xene membranes in layout (II) can be derived from layout (I) by *ad hoc* processing or massively produced *via* chemical methods (see Section 3). Scalability and massive production are addressed in Section 4.2. Stabilization is a common requisite for layouts (I) and (II) because of the air-sensitive character of the Xenes, as discussed in Section 4.2 and Fig. 4B and C, with a focus on encapsulation schemes. The achievement, stabilization, and optimization of layouts (I) and (II) can be facilitated by interface and chemical engineering using the commensurable substrate, artificially engineered template (*e.g.* heterostructures), structural phases, or processes (*e.g.* deintercalation). When integrated into a device layout based on electrical transport (as in layout III), Xenes should be standardized in terms of structural quality and electrical performance. The latter feature is closely connected to the quality of *electrical contacts*. Thus far, contacts have been made using the native substrate as an electrode element (see the case of native Ag contacts on silicene^7,101^), but more sophisticated solutions can be envisioned by taking the Xene heterostructures or hybrid structures to alleviate contact issues, such as Fermi level pinning. This is functional for having Xenes reconfigured in applications spanning energy technologies, biomedicals, flexible electronics, nanoelectronics and topological quantum technologies, and nanophotonics, as discussed in Sections 4.2 and 4.3.

In perspective, Xenes bear innovation potential in technology applications, as illustrated in Fig. 5. Three drawbacks currently limit their full exploitation, namely lack of (a) standardization, (b) viable integration schemes, and (c) killer application(s). Although epitaxial methods can yield high-quality crystals and topotactic deintercalation can be developed towards massive production, the production stage has a limited maturity in terms of product standardization, which is a necessary condition for any possible technology transfer. Processing methods, as reported in Section 4.3, represent a step closer to the deployment of the Xenes out of the surface science scope and integration into an operational platform. However, the reliability of the developed processes should be consolidated in a technology-relevant environment to assess a technology transfer. Nonetheless, substantial steps have been performed since the debut of Xenes on the stage. Nowadays, Xene membranes can be handled and made available for device testing after stabilization,^192^ and Xene nanosheets can be effectively diluted in biological solutions for biomedical applications, such as tumor and antibacterial treatment.^18,19^ Parallel to the process and technology development is the definition of a quality test standard. In this respect, Raman spectroscopy may play a pivotal role (as with graphene) because probing the Raman spectrum provides a quick and exact check of the Xene structure (both local and extensive) whenever the Xene is Raman sensitive and/or is stabilized against environmental exposure. Due to stability issues, a key condition enabling Raman quality assessment in known Xenes is the encapsulation (either heterogeneous or homogeneous). A similar issue stands out regarding functional testing, namely the figure of merits defining the target application function of the Xenes. For instance, according to biomedical applications of Xenes, it is important to note that optical absorption is the key parameter determining the ability of Xenes to localize hyperthermal phenomena aiming at tumor theranostics.^18,212^

Based on this background, the research on Xenes is progressively changing its original fundamental character towards a translation character where Xenes are made and designed for target applications. Although Xenes in flexible electronic and biomedical applications, as reported in Fig. 4E, are still at an infancy level, there is a broad consensus on the Xene potential for technology branches such as energy storage technologies, nanophotonics, and nanoelectronics,^213^ as schematically listed in Fig. 5, for each configurational layout (I)–(III) and discussed as follows.

(1) *Energy-storage technologies*: Xenes similar to borophene and silicene bear high potential as anodes for Li- and Na-ion batteries and supercapacitors because of the (theoretically) expected high charge capacitance of 954–1384 mA h g^−1^ in silicene anodes^14,214^ and 1240–1984 mA h g^−1^ in borophene^215^ compared to that of industrial graphite electrodes (372 mA h g^−1^). A flourishing consideration is nowadays given to Xenes applied to catalytic systems herein, including the hydrogen/oxygen evolution reaction and oxygen//nitrogen/CO_2_ reduction reaction,^17^ because they serve as outperforming electrode materials. Selected Xenes, such as stanene or bismuthene, are suited to design thermoelectric junctions that deviate from the Wiedemann–Franz law of conventional metals owing to the linear dispersion of the Dirac fermions. The structural anisotropy in some Xenes, such as phosphorene and tellurene, can also conveniently split the thermal and electrical conductivity towards Z*T* > 1, where Z*T* is the thermoelectric factor taken as the figure of merit to assess the thermoelectric performance.^216^

(2) *Nanophotonics*: 2D materials have an unprecedented impact on photonics and optoelectronics owing to their ability to cover the whole electromagnetic spectrum in their optical response.^217,218^ Xenes are promising in this respect because they intrinsically display variability of the electronic states according to the X constituent, the Xene phase, and functionalization. In this respect, semiconducting Xenes, such as phosphorene and tellurene, can display optical responsivity from the visible down to the far-IR regime,^219^ while silicene nanosheets on Al_2_O_3_ exhibit a characteristic quantized optical conductance.^119^ Topological Xenes, including quantum spin Hall insulators, such as germanene, stanene, and bismuthene; Dirac semimetals, such as stanene multilayer, or Weyl semiconductors, such as *n*-type tellurene, may host electronic transition induced by extremely low-energy optical excitations (in the meV regime), thus empowering long-wavelength IR and mm-wave technologies (*i.e.*, in the THz and sub-THz spectrum).^220^

(3) *Nanoelectronics and topological quantum technologies*: the field-effect transistor stands out as the device paradigm to test the electronic conduction properties of Xene at an extreme miniaturization channel scale, thereby enabling diverse functionalities, such as logic operations, radio-frequency switches, sensing, piezoelectricity, anisotropic transport, or topological features.^221,222^ Silicene was thoroughly scrutinized for its potential as a transistor active material as an ultimately shrunken channel body.^119^ Semiconducting Xenes, such as tellurene and black phosphorene, were also shown to work as active materials for memristor cells.^192^ Non-trivial topologies in the Xenes are closely related to the extent of the spin–orbit coupling, which is larger in heavier Xenes, such as germanene, stanene, plumbene, and bismuthene. This coupling eventually leads to the emergence of a quantum spin Hall state, namely a 2D topological insulating state characterized by topologically protected 1D edge states bearing nondissipative transports at the physical borders (*e.g.* at the perimeter of a Xene nanoribbon) and a gap opening in the 2D body (*e.g.* inside the Xene nanoribbon). Recently, localized edge states stemming from such non-trivial topology were observed by STM of bismuthene on SiC^87^ and on germanene released from PtGe_2_.^60,186^ In the latter case, an electrical field-dependent topological transition was reported, which is preparatory to engineering a topological bit, namely a logic (binary) state determined by the topological state, and more generally to further up a paradigm shift to a topological quantum computation scheme.^185^ All along this line, heavy Xenes, such as stanene, proved to be building blocks for fabricating new topological and quantum materials at the 3D level, taking the case of superconductivity in stanene trilayers as an example.^12^ The evolution of Xene towards the topological matter is still an open field with possible outstanding implications in quantum computing technologies (*e.g.*, see the achievement of Majorana fermion systems by coupling topological Xene with a superconducting material), catalysis, and photonics.^223^

Despite some preliminary facts (either theoretical outcomes or experimental evidence), a lab-to-fab transition for Xenes is still hurdled by the lack of maturity in the release of technology transfer protocols and in the uncertainty of clearcut killer applications justifying a substantial investment in the large-scale production of (selected) Xenes. This is the key issue for which the impact of Xene on a technological background will be assessed in a few years. However, in a long-term materials research framework, it is clear that Xenes represent an extreme manipulation of the elements to create artificial material platforms and design new physical properties. The number of Xenes is probably yet to be complete, but in our view, it is likely to envisage Xenes as a building block of multifunctional materials with pre-configurable properties on demand (*e.g.*, in Xene heterostructure and hybrid structures) or as a template to promote non-conventional 3D materials.

## Data availability

No primary research results or new data were generated or analyzed as part of this review. The elaboration of previously published data presented in the figures and tables of this manuscript will be made available by uploading the accepted manuscript to the public repository “Zenodo” with an associated DOI, in full compliance with the publisher's embargo conditions. Additionally, engagement with the Royal Society of Chemistry will facilitate the open-access publication of the accepted manuscript.

## Conflicts of interest

There are no conflicts to declare.
